# Engineering crops of the future: CRISPR approaches to develop climate-resilient and disease-resistant plants

**DOI:** 10.1186/s13059-020-02204-y

**Published:** 2020-11-30

**Authors:** Syed Shan-e-Ali Zaidi, Ahmed Mahas, Hervé Vanderschuren, Magdy M. Mahfouz

**Affiliations:** 1 Plant Genetics, TERRA Teaching and Research Center, Gembloux Agro-Bio Tech, University of Liège, Gembloux, Belgium; 2 Laboratory for Genome Engineering and Synthetic Biology, Division of Biological Sciences, 4700 King Abdullah University of Science and Technology, Thuwal, 23955-6900 Saudi Arabia; 3 Laboratory of Tropical Crop Improvement, Division of Crop Biotechnics, Biosystems Department, KU Leuven, Leuven, Belgium

## Abstract

To meet increasing global food demand, breeders and scientists aim to improve the yield and quality of major food crops. Plant diseases threaten food security and are expected to increase because of climate change. CRISPR genome-editing technology opens new opportunities to engineer disease resistance traits. With precise genome engineering and transgene-free applications, CRISPR is expected to resolve the major challenges to crop improvement. Here, we discuss the latest developments in CRISPR technologies for engineering resistance to viruses, bacteria, fungi, and pests. We conclude by highlighting current concerns and gaps in technology, as well as outstanding questions for future research.

## Introduction

Global population is growing at an alarming rate and is expected to increase by one quarter in the next 30 years, to reach 10 billion [1, 2]. Meanwhile, agricultural land area per capita, defined as the sum of arable land, permanent cropland, permanent meadows, and pastures, is declining every year [3]. Climate change is making the situation even more grim, with global temperatures expected to rise 2 °C by the year 2050. In Europe, for example, a recent study estimated that, as compared to the year 2000, summer and winter temperatures would increase by 3.5 °C and 4.7 °C, respectively, by that time [4]. This temperature shift will significantly affect the patterns of pathogen infection, making crop diseases more severe and less predictable [5]. Rising temperature is not the only threat to agriculture from climate change, as rising sea levels also exacerbate the scarcity of arable land; one such event has already caused massive locust swarms that severely damaged crops across East Africa, Asia, and the Middle East in 2020 [6]. With population increasing while agricultural land area decreases and crops experience constant threats from climate change, a vital route toward maintaining food security is the development of climate-resilient crops.

To cope with these challenges, scientists and plant breeders have been consistently working to develop new crop varieties that are not only high yielding, but also resistant to relevant abiotic stresses such as drought, salinity, and flooding, and biotic stresses such as insects and pathogens. As a result, the average crop yield per hectare of agricultural land has increased significantly since the Green Revolution of the 1950s and 1960s [3, 7]. The developing world has witnessed an extraordinary period of growth in food crop productivity during the past few decades, despite increasing land scarcity and rising land values. While populations more than doubled, cereal crop production tripled during this period, with only a 30% increase in the area of land under cultivation. This pattern underscores the importance of improved crop varieties in meeting rising food demands in the past. It must be noted here that the productivity boost during the Green Revolution was due not only to improved crop varieties but also to strong investment in crop research, infrastructure, and market development and appropriate policy support, highlighting the importance of these processes for the future [7]. Nevertheless, it can be concluded that improved food crop varieties will make it possible to address the challenges of food scarcity and mitigate the effect of climate change on agriculture.

## Production of engineered crops via new plant breeding technologies

Crop varieties have conventionally been developed by farmers and crop breeders using basic techniques such as the selection of plants with desirable characteristic for propagation. Modern plant breeding techniques added marker-assisted selection and genetic modification to the crop improvement toolkit. These methods have been reviewed elsewhere [8, 9]. Briefly, a genetically modified (GM) crop variety is developed by (1) identification of a piece of DNA that confers the trait of interest, for example, a gene responsible for virus resistance; (2) cloning of the DNA into the carrier or vector plasmid; (3) delivery of the DNA to the target plant; and (4) generation of modified plants with the desired trait, e.g., virus resistance. GM crop production has been controversial mainly because of fear-based agricultural policies driven by limited public understanding, ineffective information sharing by scientists, and inaccurate portrayals by NGOs and anti-GM lobbyists [10]. Apart from social and economic concerns such as ownership, stewardship, product regulation, and market development, one major concern related to GM crops is the extensive use of certain agrochemicals (such as glyphosate) in conjunction with herbicide-tolerant GM crop varieties and the retention of antibiotic-resistance genes from the production pipeline in the GM variety. These concerns have led to the enactment of strict regulations for GM crops, which not only make the end products expensive but also slow the delivery of new varieties to farmers, making it more difficult for breeders to produce varieties suited to current threats to crops.

While society remains divided over the use of GM crops, new plant breeding technologies (NPBTs) have recently emerged as alternative approaches to speed up the introduction of improved traits. NPBTs include precision genome-modification platforms such as the clustered regularly interspaced short palindromic repeat (CRISPR)/CRISPR-associated protein (Cas) and transcription activator-like effector nuclease (TALEN) methodologies [11]. In addition to genome editing, NPBTs include technological advances that shorten the breeding cycles and accelerate crop research, such as speed breeding [12, 13] and next-generation genotyping [14] and phenotyping platforms [15]. For some important crops that have a flowering behavior difficult for breeding (such as cassava, *Manihot esculenta*) or are sterile (such as banana, *Musa acuminata*), genome editing provides an efficient and robust breeding approach, given the alternative breeding approaches are either significantly inefficient or not applicable [16].

Genome-editing NPBTs differ from conventional GM methods in various ways. For example, TALENs and CRISPR-Cas can be used for precise genetic manipulation without introducing exogenous DNA such as antibiotic-resistant genes, thus eliminating the fear that foreign DNA may be present in the final product [17]. Whereas classical GM crop production requires the insertion of foreign DNA (transfer DNA, or T-DNA, from *Agrobacterium* species), some genome-editing protocols do not require T-DNA insertion, such as CRISPR via a ribonucleoprotein (RNP) complex or via virus-based DNA replicons to induce precisely targeted edits in the crop plant DNA [18–20]. Further elaborations of this methodology, including protoplast delivery of preassembled CRISPR-Cas RNPs, transient expression of programmable nucleases through agroinfiltration, and site-specific integration of a CRISPR array in other chromosomal locations followed by removal via segregation, have paved the way toward developing transgene-free CRISPR plants [17, 21, 22].

These recent developments in NPBTs make it possible for new food products to reach the market quickly at affordable prices. Recent examples of such products include browning-resistant mushrooms [23], high-amylopectin waxy corn (*Zea mays*) [24], and false flax (*Camelina sativa*) with enhanced omega-3 oil [25]—all of which were developed using CRISPR and approved by the US Department of Agriculture (USDA) in record time. In summary, the latest developments in NPBTs make them excellent tools with which to produce the crops of the future, by making it possible to address concerns related to GM crops and because of their precision, robustness, and timely regulation.

## CRISPR-mediated genome editing: the evolution of site-specific nucleases

Genome editing uses site-specific nucleases (SSNs), which can be designed to bind and cleave a specific nucleic acid sequence, introducing double-stranded breaks (DSBs) at or near the target site [26]. There are four major classes of SSNs: meganucleases, zinc-finger nucleases (ZFNs), TALENs, and Cas proteins [26, 27]. These SSNs have significant potential for plant breeding, as they provide multifaceted mechanisms to modulate host genome structure and function, including gene knock-out, knock-in, and stacking, targeted mutagenesis, and modulation of translation. SSNs offer significant economic advantages and save time compared to conventional plant breeding approaches, which can take up to 10 years for variety development [28].

Notably, the CRISPR/Cas system has emerged as the leading, ground-breaking SSN and, although its utility for plant genome editing was first demonstrated only in 2013 [29–31], its applications in plants have increased rapidly compared to other NPBTs. Research using CRISPR has introduced important agricultural traits including heat, cold, and herbicide tolerance; viral, bacterial, and fungal resistance; and increased grain size and weight into many economically important crops, such as rice (*Oryza sativa*), wheat (*Triticum aestivum*), maize (*Z*. *mays*), tomato (*Solanum lycopersicum*), potato (*Solanum tuberosum*), tobacco (*Nicotiana tabacum*), cotton (*Gossypium* spp.), soybean (*Glycine max*), and brassicas [28]. Importantly, several groups have recently accomplished those genome alterations using transgene-free systems.

The working principle of CRISPR-Cas9 has been well explained in several recent reviews [28, 32]. Briefly, CRISPR-Cas9 is a type ΙΙ adaptive immune system, identified initially in *Streptococcus pyogenes*, that provides prokaryotes with defenses against invading phages [33]. This system, the simplest CRISPR type, relies on the induction of site-specific DSBs in the DNA of the invading virus. These DSBs consequently induce a cellular DNA-repair mechanism either via non-homologous end-joining (NHEJ), which leads to imprecise repair, or via homology-directed repair (HDR), which leads to precise repair. Repair introduces insertions or deletions (indels) in the invader virus DNA and leads to a dysfunctional virus, providing natural defense to the bacteria against viruses.

In an engineered system, a CRISPR locus transcribes a short CRISPR RNA (crRNA) that hybridizes to a complementary sequence on the targeted genome (protospacer) adjacent to the protospacer-associated motif site (PAM). In the case of *S*. *pyogenes*, the PAM is a trinucleotide sequence, canonically 5′-NGG-3′, that is essential for Cas9 to selectively recognize and bind targeted viral DNA [34]. Next, a trans-activating RNA (tracrRNA) binds the crRNA to process the mature guide RNA (gRNA) and pair up with the endonuclease Cas9 and RNase III to form the Cas9 complex. Once the gRNA binds the complementary target site, it guides the Cas9 nuclease to generate a DSB three nucleotides upstream of the PAM site on the target nucleic acid [35]. CRISPR-Cas9 thus has the potential to induce precise, site-specific genome editing through the delivery of synthetic single guide (sg) RNAs designed to guide Cas9-mediated cleavage at targeted sites [32].

With the advances in CRISPR-Cas, substantial, ongoing work is using this technique to improve crops through metabolic engineering and regulation of host genes. Several new CRISPR systems, such as Cas12 [36], Cas13a [37, 38], Cas13b [39], and fCas9 [40], are currently in the pipeline. These advances have led to the development of new genome-editing applications that include deactivated Cas9 (dCas9) [41, 42], RNA-processing Cas9 (RCas9) [37, 43], and Cas9 fusion proteins such as Cas9-cytidine deaminase fusion [44], offering scientists a wide range of CRISPR-based applications.

## Approaches to design disease-resistant plants with CRISPR technologies

Recent advances in CRISPR technology have enabled scientists to develop a broad range of CRISPR variants with different applications, including the modifications described above. In this section, we focus on the CRISPR applications that have been successfully used to engineer disease-resistant plants.

### Gene disruption via indels in coding sequences

**Fig. 1:**
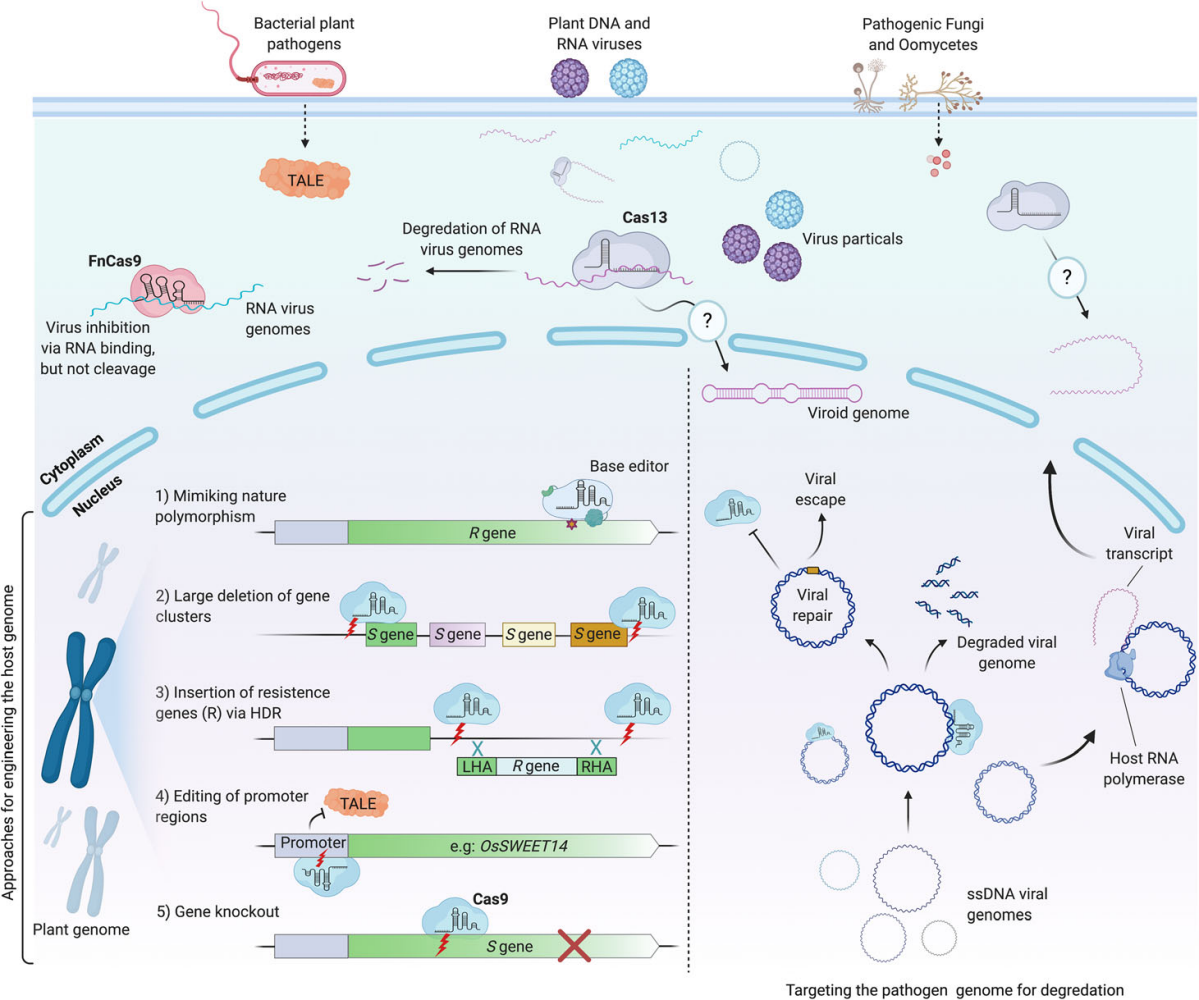
Application of CRISPR/Cas-based technologies for engineering disease-resistant plants. CRISPR technology, most widely with the Cas9, can be applied to achieve precise genome editing of the plant genome to develop resistance against various pathogens. CRISPR/Cas9 can be used to disrupt plant susceptibility (*S*) genes by targeting coding regions to knock out these genes, or to alter sequences of promoter regions (for example, pathogen promoter’s effector-binding site), precluding pathogen effector binding to the promoter and thus disrupting plant susceptibility. In addition, the ability of performing Cas9-mediated multiplex targeting can facilitate the chromosomal deletion of *S* gene clusters, generating long-term resistance to the target pathogen. Homology-directed repair (HDR) mediated by Cas9 can be used to introduce resistance (*R*) genes against pathogens in cases where the plant-pathogen interaction (and *S* genes) is not well studied. To develop pathogen resistance without disrupting or replacing whole genes, base-editors or Cas9 technology (via synthetic directed evolution under the biotic selective pressure) can be applied to achieve specific mutations (biomimicking) or evolution of genes resistant to pathogens of interest. Apart from utilizing CRISPR technologies for plant genome engineering to develop disease-resistant plants, the native function of CRISPR can be mimicked to directly target and interfere with the genomes of pathogens of interest without affecting plant genome. For example, CRISPR can interfere with DNA genomes of pathogens, such as DNA viruses, through DNA-targeting CRISPR systems, including Cas9. CRISPR systems can also be used to target and disrupt pathogen’s RNA genomes (or RNA transcript of pathogens with DNA genomes) through RNA-targeting CRISPR systems, such as Cas13 and FnCas9

This is the most common application of the CRISPR-Cas9 system. It takes advantage of the error-prone behavior of the cellular NHEJ DNA-repair machinery. The result is an insertion or deletion (indel) of one or more nucleotides at the sgRNA-guided site, which introduces a frameshift mutation and disrupts gene production (Fig. 1). This technology has been used in several crops, including essential cereals such as rice and wheat, to introduce traits of interest [28]. In the context of disease resistance, this technology has been used to engineer resistance by disrupting a plant susceptibility (*S*) gene, which alters the plant-pathogen interaction, leading to reduced pathogen fitness on the host plant [45]. A remarkable example of this application was the use of CRISPR-Cas9 to introduce indels affecting eukaryotic translation initiation factor 4E proteins (eIF4Es), which successfully induced resistance against multiple RNA viruses in *Arabidopsis* and cucumber (*Cucumis sativus*) [46, 47].

### Gene disruption via indels in promoter regions

A similar approach can be used to introduce indels in the promoter region instead of the coding region of a plant gene. CRISPR-mediated promoter editing can be applied in two ways: to disrupt the promoter sequence, with the aim of blocking gene expression entirely, or to disrupt an effector-binding site, with the aim of disrupting plant susceptibility by preventing a pathogen effector binding to the promoter. The latter approach has been used to modify the promoter of the rice sugar transporter gene *OsSWEET14*, breaking the connection with the effector from a bacterial blight pathogen and thus leading to blight resistance (Fig. 1) [48–51]. In addition to promoter editing, CRISPR has been demonstrated to alter the gene regulation by targeting upstream open reading frame (ORF) regions and editing *cis*-regulatory elements [52].

### Gene deletion via multiplex sgRNAs

In CRISPR systems, multiple sgRNAs can be used to introduce multiple DSBs at precise locations in the target genome. For instance, two sgRNAs binding before the start codon and after the stop codon of the gene of interest will produce DSBs at the respective locations. These DSBs then result in the removal of the DNA fragment containing the gene of interest, before the cellular repair NHEJ machinery repairs the DSBs. Because sgRNAs can be designed at any genomic region containing an appropriate PAM trinucleotide sequence, this approach can be and has been used to delete large chromosomal fragments as well as individual genes [53, 54]. This is further facilitated by the development of a rationally designed Cas9, SpCas9-NG, that can recognize NG PAMs, a relaxed stringency compared with the typical NGG PAMs [55]. In the context of engineered pathogen resistance, gene clusters make this approach particularly useful. In *S* gene clusters, where multiple *S* genes reside in adjacent chromosomal locations [56], deleting the chromosomal fragment is likely to generate long-term resistance to the target pathogen (Fig. 1).

### Gene insertion via homology-directed repair

The aforementioned CRISPR techniques can be used to generate disease resistance through alteration of *S* gene(s). However, all plant proteins, including the products of *S* genes, are important and mostly multifunctional; disrupting these proteins thus comes with costs to plant health and/or productivity. There are alternate approaches, such as the aforementioned *cis*-regulatory element and promoter editing, to alter the gene expression instead of gene disruption, but it is often necessary to utilize resistance (*R*) genes against pathogens in cases where the plant-pathogen interaction is not well studied, and the *S* genes have not been extensively explored. In such cases, the CRISPR toolkit can be used for *R* gene insertion.

CRISPR-mediated gene insertion works via an alternative route that operates after Cas9 has produced the sgRNA-dictated DSB; this route utilizes the cellular HDR, rather than NHEJ, machinery. A delivery fragment, containing an *R* gene surrounded by sequence homologous to the DSB ends, is supplemented with Cas9 and the sgRNAs. This cassette guides the HDR-mediated insertion of the *R* gene between the two DSB sites (Fig. 1). This strategy has been used to introduce one or more genes at precise genomic locations [57]. However, the efficiency of HDR in plants is very low [58], and although new strategies to improve this are under development, currently, this makes gene insertion in plants challenging to implement [59].

In addition to generating disease resistance, CRISPR-mediated gene insertion can be used to study important *S* gene functions. To demonstrate this, Wang et al. used HDR in rice to incorporate green fluorescent protein (GFP) fused in frame with the glutathione S-transferase loci [60]. This application has the potential to be used in studying *S* gene functions, such as protein localization and the spatiotemporal regulation of *S* gene expression. Host proteins playing key roles in pathogenicity can be tagged with GFP directly in the genome in order to study their expression and localization during infection. However, such protein modifications can alter their expression and localization, demonstrating the limitations of this approach.

### Biomimicking via promoter, allele, or gene replacement

The concerns surrounding the introduction of foreign DNA into crop products mean that the use of CRISPR for gene insertion is likely to be subjected to lengthy regulatory processes and potentially consumer rejection. To overcome this challenge, CRISPR offers an alternative approach that works on the principle of biomimicking. It has been known for decades that native species and wild relatives of cultivated crops constitute a rich gene pool, especially for resistance against biotic and abiotic stresses [61]. Several recent studies have identified many *R* genes in the wild relatives of cultivated species and demonstrated the successful transfer of resistance via the identification of an *R* gene and its transfer to the cultivated crop species [62, 63]. CRISPR can be used to replace the faulty or poor-performing *R* gene in a cultivated crop variety with the functional *R* gene from a disease-resistant native variety via multiplexed HDR methodology.

Biomimicking refers here to the introduction of CRISPR-mediated mutations in such a way that the sequence of the target gene is converted to the sequence from a disease-resistant variety. Thus, instead of replacing the whole gene, the researcher introduces only the specific mutations associated with the disease resistance trait, assuming that the nucleotide differences between the gene of interest in the cultivated and wild varieties are not otherwise significant to plant viability and productivity (Fig. 1). This has been mainly achieved by utilizing a CRISPR system designed for targeted nucleotide modification. A fusion of a nuclease-dead Cas9 or nickase with cytidine deaminase can target point mutagenesis with high precision, and this approach has been successfully used in several species for gene modification [64]. The same approach has been used to introduce the N176K substitution encoded by the eIF4E virus-resistance allele (eIF4E1) of *Pisum sativum* into the *Arabidopsis EIF4E1* gene to generate *Arabidopsis* plants resistant to *Clover yellow vein virus* (ClYVV) [65]. Recent studies have established the use of synthetic directed evolution to evolve gene variants resistant to splicing inhibitors and herbicides [66–68]. The same approach may be used under the biotic selective pressure to evolve gene variants conferring resistance to selected pathogens.

## CRISPR-mediated disease resistance: what has been achieved so far?

CRISPR technology and its variants have been used for applications in plant science ranging from the study of gene function and protein localization to the introduction of desired traits such as drought tolerance and increased grain size and number. These interesting applications have been covered in recent review articles [26, 28]; here, we focus on CRISPR-mediated engineering of plant disease resistance. Plant diseases are mainly caused by infection with one or more of the main categories of plant pathogens: i.e., viruses, bacteria, and fungi. CRISPR technology has been used to engineer resistance against all these major plant pathogen classes (Table 1).

**Table 1:** A summary of the studies on CRISPR-mediated plant disease resistance

Pathogen type	Plant(s)	Desired modification	Targeted DNA/RNA	Targeted pathogen(s)/disease(s)	Results	Reference
m	*Arabidopsis*	Virus RNA genome disruption	Virus RNA genome	*Turnip mosaic virus*	Indels in virus RNA	[69]
	*N*. *benthamiana*	Virus RNA genome disruption	Virus RNA genome	*Turnip mosaic virus*	Indels in virus RNA	[37]
	Rice, *N*. *benthamiana*	Virus RNA genome disruption	Virus RNA genome	*Southern rice black-streaked dwarf virus*, *Tobacco mosaic virus*	Reduction in virus levels and disease symptoms	[70]
	*Arabidopsis*, *N*. *benthamiana*	Virus RNA genome disruption	Virus RNA genome	*Cucumber mosaic virus*, *Tobacco mosaic virus*	Reduction in virus levels and disease symptoms	[71]
	*N*. *benthamiana*	Virus DNA disruption	Virus DNA Rep, IR, and Cp	*Beet curly top virus*, *Merremia mosaic virus*, *Tomato yellow leaf curl virus*	Indels in virus DNA	[72]
	*N*. *benthamiana*	Virus DNA disruption	Virus DNA and satellite sequences	*Cotton leaf curl Kokhran virus*, *Tomato yellow leaf curl Sardinian virus*, *Tomato yellow leaf curl virus*, *Merremia mosaic virus*, *BCTV-Logan*, *BCTV-Worland*	Indels in virus DNA	[73]
	*N*. *benthamiana*	Virus DNA disruption	Virus DNA Rep A/Rep and LIR	*Bean yellow dwarf virus*	Indels in virus DNA, resistance to virus	[74]
	*Arabidopsis*, *N*. *benthamiana*	Virus DNA disruption	Virus DNA Rep, IR, and CP	*Beet severe curly top virus*	Indels in virus DNA, resistance to virus	[75]
	Tomato, *N*. *benthamiana*	Virus DNA disruption	Virus DNA Rep, IR, and Cp	*Tomato yellow leaf curl virus*	Indels in virus DNA, resistance to virus	[76]
	*N*. *benthamiana*	Virus DNA disruption	Multiplex editing at Rep and IR	*Cotton leaf curl Multan virus*	Significantly low virus accumulation and decreased disease symptoms	[77]
	Cassava	Virus DNA disruption	AC2 and AC3	*African cassava mosaic virus*	Indels in virus DNA but no virus resistance	[78]
	*N*. *benthamiana*	Virus DNA disruption	Multiplex editing at virus DNA Rep, IR, and Cp	*Chilli leaf curl virus*	Significantly low virus accumulation and decreased disease symptoms	[79]
	Banana	Virus DNA disruption	Virus sequences in the host plantain genome	*Endogenous banana streak virus*	75% of pl0ants remain asymptomatic	[80]
		Biomimicking^a^	*Eif4e1*	*Clover yellow vein virus*	Reduced virus accumulation	[65]
	Rice	Biomimicking^a^	*Eif4g*	*Rice tungro spherical virus*	Resistance to virus	[81]
	Cassava	Gene disruption	*nCBP-1*, *nCBP-2*	*Cassava brown streak disease*	Suppressed disease symptoms	[82]
	*Arabidopsis*	Gene disruption	*EIF4E*	*Turnip mosaic virus*	Resistance to virus	[47]
	Cucumber	Gene disruption	*eIF4E*	*Cucumber vein yellowing virus* (ipomovirus), *Zucchini yellow mosaic virus*, and *Papaya ring spot mosaic virus-W* (potyviruses)	Resistance to three viruses	[46]
Fungus	Tomato	Gene disruption	Multiplex gRNA at *Pmr4*	Powdery mildew caused by *Oidium neolycopersici*	Significant reduction in mildew symptoms	[83]
	Tomato	Gene disruption	*SlMapk3*	*Botrytis cinerea*	Increased resistance to *B*. *cinerea*	[84]
	Tomato	Gene disruption	*Solyc08g075770*	Fusarium wilt	Tolerance to fusarium wilt	[85]
	Rice	Gene disruption	Single and multiplex gRNA at *OsERF922*	Rice blast caused by *Magnaporthe oryzae*	Significantly decreased blast lesions	[86]
	Grape	Gene disruption	*VvWRKY52*	*B*. *cinerea*	Increased resistance to *B*. *cinerea*	[87]
	Tomato	Gene disruption	*SlMlo1*	Powdery mildew	Resistance to powdery mildew	[88]
	Banana	Gene insertion	*RGA2*, *Ced9*	Fusarium wilt caused by *Fusarium oxysporum* f. sp. *cubense* tropical race 4 (TR4)	Significant reduction in disease	[89]
	Rice	Gene disruption	*OsMPK5*	Fungal (*Magnaporthe grisea*) and bacterial (*Burkholderia glumae*) pathogens	Indels in the target; resistance not confirmed	[90]
	Grape	Gene disruption	*Mlo-7*	Powdery mildew	Indels in the target; resistance not confirmed	[91]
	Wheat	Gene disruption	*TaMlo-A1*, *TaMlo-B1*, and *TaMlo-D1*	Powdery mildew	High tolerance to powdery mildew	[92]
	Wheat	Gene disruption	*TaMlo*	Powdery mildew	Indels in the target; resistance not confirmed	[30]
	Wheat	Gene disruption	*TaEdr1* (three homologs)	Powdery mildew	Resistance to powdery mildew	[93]
Bacteria	Rice	Gene disruption	*OsSWEET13*	Bacterial blight caused by *Xanthomonas oryzae* pv. *Oryzae* (Xoo)	Resistance not confirmed	[94]
	Rice	Gene disruption	*OsSWEET11*	Bacterial blight	Enhanced resistance to Xoo	[95]
	Rice	Gene and promoter disruption	TALE-binding elements (EBEs) in *OsSWEET13* promoter, *OsSWEETT11*, and *OsSWEEt14* genes	Bacterial blight caused by Xoo	Broad-spectrum resistance against multiple Xoo strains	[50]
	Rice	Promoter disruption	*OsSWEET11*, *OsSWEET13*, and *OsSWEET14*	Bacterial blight	Increased resistance to bacterial blight; confirmed in field trials	[51]
	Apple	Gene disruption	*DIPM-1*, *DIPM-2*, and *DIPM-4*	Fire blight disease (caused by *Erwinia amylovora*)	Indels in the target; resistance not confirmed	[91]
	Rice	Promoter disruption	*OsSWEET11*, *OsSWEET14*	Bacterial blight	Indels in promoter; disease resistance not confirmed	[96]
	Tomato	Gene repair	*Jaz2*	Bacterial speck disease caused by *Pseudomonas syringae* pv. *tomato* DC 3000	Resistance to bacterial speck disease	[97]
	Tomato	Gene disruption	*Dmr6*	*Pseudomonas syringae*, *Phytophthora capsici*, and *Xanthomonas* spp.	Resistance to *P*. *syringae*, *P*. *capsici*, and *Xanthomonas* spp.	[98]
	Grapefruit	Promoter disruption	*CsLOB1*	Citrus canker	Significantly reduced canker symptoms	[99]
	Wanjincheng orange	Promoter disruption	*CsLOB1*	Citrus canker	Disease severity decreased by 83.2–98.3%	[100]
Oomycete	Papaya	Gene disruption	*PpalEPIC8*	*Phytophthora palmivora*	Increased resistance against *P*. *palmivora*	[101]
	*Theobroma cacao*	Gene disruption	*TcNPR3*	*Phytophthora tropicalis*	Increased resistance against *P*. *tropicalis*	[102]

^a^Biomimicking refers here to the introduction of CRISPR-mediated mutations in such a way that the sequence of a target gene in disease-susceptible variety is converted to the sequence from a disease-resistant variety. Thus, instead of replacing the whole gene, the researcher introduces only the specific mutations associated with the disease resistance trait, assuming that the nucleotide differences between the gene of interest in the cultivated and wild varieties are not otherwise significant to plant viability and productivity

### CRISPR-mediated resistance against plant viruses: targeting virus genomes

Plant viruses are usually managed through improved agricultural practices and the use of virus-resistant crop varieties. Most studies of CRISPR-mediated pathogen resistance in plants have involved resistance to viruses. These studies have demonstrated two main ways to engineer virus resistance: (1) directly targeting virus genome and (2) targeting plant *S* genes crucial for the development of the viral disease (Fig. 1) [45].

The earliest work on engineered virus resistance used the first approach of directly targeting the virus genome inside plant cells [103]. In two independent studies, Ali et al. targeted multiple single-stranded DNA (ssDNA) geminiviruses: *Beet curly top virus*, *Merremia mosaic virus*, *Tomato yellow leaf curl virus*, and *Cotton leaf curl Kokhran virus* with its helper betasatellite cotton leaf curl Multan betasatellite, and successfully demonstrated virus targeting by the sgRNA [72, 73, 76]. Within the viral genome, three sites were targeted, two encoding replication-associated protein and capsid protein, and one an intergenic region. Targeting these sites introduced indels in virus genome consequently leading to the lower virus titer and significantly reduced disease symptoms. Baltes et al. and Ji et al. demonstrated similar results, targeting the geminiviruses *Bean yellow dwarf virus* and *Beet severe curly top virus*, respectively [74, 75]. Apart from CRISPR-mediated targeting of the viruses, these studies demonstrated limited success in achieving virus resistance in permanent transgenic systems. A similar approach targeting the endogenous *Banana streak virus* (eBSV) within the genome of plantain banana (*Musa* spp.) demonstrated that 75% of the edited events remained asymptomatic compared to the non-edited plants [80].

Direct targeting of virus genomes via CRISPR is a means to engineer resistance against RNA viruses as well. A similar approach targets the viral RNA, instead of DNA, and uses the Cas9 RNA-binding variants, instead of DNA-binding Cas9. Zhang et al. developed *Nicotiana benthamiana* and *Arabidopsis* plants expressing a FnCas9 targeting *Cucumber mosaic virus* and *Tobacco mosaic virus* (TMV) and observed a significant reduction in virus accumulation and reduced symptom development (Fig. 1) [71]. They later utilized a similar strategy to produce resistance to *Southern rice black-streaked dwarf virus*, an RNA virus targeting rice [70]. In two independent studies, Aman et al. also used an RNA-targeting LwaCas13a (previously known as C2c2) to interfere with the RNA genome of *Turnip mosaic virus* (TuMV) and demonstrated successful virus RNA targeting in both *N*. *benthamiana* [37] and *Arabidopsis* [69]. Although LwaCas13a showed moderate targeting efficiency against TuMV in Aman et al. studies, these studies showed the great potential of using Cas13 systems in engineering plant immunity against RNA viruses. This led Mahas et al. to further expand the Cas13-mediated virus immunity by screening various Cas13 variants and identifying Cas13d system (CasRx) as the most robust Cas13 ortholog to target and interfere with different RNA viruses, including TMV, *Potato virus X* (PVX), and TuMV (Fig. 1) [104]. The impressive efficiency of Cas13 for targeting and interfering with RNA viruses in plants could be exploited to potentially provide immunity against the economically significant plant-infecting viroids. Whether Cas13 could successfully target and degrade circular, highly compact and secondary structure-rich RNA viroid genomes remain to be seen [105].

Ali et al. highlighted a potential problem with the system by demonstrating the possibility of virus escape from the CRISPR-edited plants [73]. Virus escape was later confirmed by Mehta et al., whose attempt to engineer resistance against *African cassava mosaic virus* in permanent transgenic cassava (*Manihot esculenta*) lines failed to establish resistance [78]. Further investigation revealed that 33–48% of edited virus genomes carried a conserved single-nucleotide mutation that conferred resistance to CRISPR-Cas9 cleavage and generated virus escape mutants (Fig. 1) [78]. A proposed strategy to counter this limitation is the use of a multiplex CRISPR system to introduce multiple DSBs in virus DNA, which should be less prone to NHEJ-repaired functional virus mutations and, in turn, to the development of escape mutants [106]. A recent study has indeed demonstrated the targeting of a geminivirus, *Chilli leaf curl virus*, at multiple sgRNA target sites and a significant reduction in viral DNA accumulation [79]. Subsequent transient assays confirmed a significant decrease in viral DNA accumulation and disease symptoms [79]. Similar results were obtained by Yin et al., who used a multiplex CRISPR system to target *Cotton leaf curl Multan virus* at multiple genomic sites (Rep and IR) and achieved successful resistance against virus in transgenic *N*. *benthamiana* plants [77]. Further studies in crop plants and tests in field trials will be needed to validate these findings. Although alternative strategies such as multiplex editing are aimed at addressing this, the potential for viral escape and the evolution of CRISPR-resistant viruses remain the biggest concerns with this approach and limit the applications of direct viral-genome-targeting CRISPR technology [106, 107].

### CRISPR-mediated resistance against plant viruses: targeting plant *S* gene(s)

CRISPR-mediated targeting of *S* gene/s avoids the limitations associated with direct viral genome targeting, as with this approach, the sgRNA targets the plant gene instead of the viral genome, which is more prone to evasion because of its high copy number and high recombination rate. Moreover, *S* gene disruption can be performed following transgene-free CRISPR protocols. Thus, when *S* genes are known and well characterized, CRISPR-mediated targeting of *S* genes appears to be a better approach to engineer viral immunity.

The *S* genes most widely targeted in CRISPR-mediated engineering of virus resistance are the e*IF4E* genes, which encode cap-binding proteins essential for the cellular infection cycle of various RNA potyviruses. The potyviral 5′-terminal protein VPg interacts with eIF4Es, and blocking this interaction triggers immunity against potyviruses in various plant species. In two independent studies, CRISPR-mediated *eIF4E* targeting yielded successful resistance against multiple viruses, including TuMV, *Cucumber vein yellowing virus*, *Zucchini yellow mosaic virus*, and *Papaya ring spot mosaic virus-W*, in *Arabidopsis* and cucumber [46, 47]. Later studies targeting eIF4E isoforms in other important crop species produced broad-spectrum virus resistance in a variety of hosts. Gomez et al. targeted the eIF4E isoforms in cassava, novel cap-binding protein-1 (nCBP-1) and nCBP-2, and showed that this significantly suppressed the symptoms of *Cassava brown streak virus* disease [82]. Furthermore, a biomimicking approach generated CRISPR-mediated mutations in *Arabidopsis* eIF4E1 that converted its sequence to match the *P*. *sativum* eIF4E virus-resistance allele, successfully introducing resistance against ClYVV [65]. Likewise, Macovei et al. demonstrated resistance in rice against rice *Tungro spherical virus* through biomimicking of *eIF4G* alleles [81]. In summary, multiple CRISPR systems have utilized *S* genes to achieve virus resistance in a number of crop species.

### CRISPR-mediated resistance against bacteria

The most successful example of CRISPR-mediated introduction of bacterial resistance in crops is by using *OsSWEET* gene/s to trigger immunity against bacterial blight caused by *Xanthomonas oryzae* pv. *oryzae*. The rice *SWEET* clade III family contains the genes *OsSWEET11*, *OsSWEET13*, and *OsSWEET14*, which encode sugar transporters mediating glucose and sucrose export; SWEET gene induction by transcription activator-like effectors (TALEs, the bacterial proteins used to develop TALENS) triggers sugar release into the apoplast, providing a nutrient source for the pathogen, and thus, these genes act as *S* genes. Genome editing via TALENs had already demonstrated that rice bearing one or more *OsSWEET* knock-outs shows resistance to bacterial blight [48, 49]. Further studies identified the TALE-binding elements (EBEs) in the *OsSWEET* promoters as key virulence factors in recessive OsSWEET-mediated resistance [50, 94]. Recently, a comprehensive study introduced mutations into the promoters of all three *OsSWEET* genes via CRISPR-Cas9 and reported that this resulted in broad-spectrum resistance to bacterial blight (Fig. 1) [51].

In tomato (*Solanum lycopersicum*), bacterial speck disease is caused by *Pseudomonas syringae* pv. *tomato* DC 3000, which produces coronatine that, with the co-receptor JAZ2, stimulates stomata opening and facilitates leaf colonization by the bacteria. CRISPR-Cas9 has been used to edit *SlJAZ2* to produce a dominant allele encoding a variant of JAZ2 that lacks the C-terminal Jas domain, which prevents stomatal reopening and provides resistance to bacterial speck disease [97]. Another *Arabidopsis* gene, *DMR6* (*DOWNY MILDEW RESISTANT 6*), is strongly associated with salicylic acid regulation and, in turn, with pathogen infection in plants. CRISPR has been used to target its tomato ortholog, *SlDmr6-1*, conferring broad-spectrum resistance against multiple pathogens including *P*. *syringae*, *Phytophthora capsici*, and *Xanthomonas* spp. [98].

One of the most economically important bacterial diseases for which CRISPR provides a resistance solution is citrus canker. This infection, caused by *Xanthomonas citri* subsp. *citri*, is considered among the most destructive diseases of citrus, causing yield losses in citrus-growing regions worldwide. The main TALE of the bacterium, PthA4, specifically binds to the effector-binding element in the promoter of a citrus canker-susceptibility gene, *LATERAL ORGAN BOUNDARIES 1* (*CsLOB1*), and activates its expression to favor citrus canker development [99]. Two independent studies have demonstrated that CRISPR-mediated editing of *CsLOB1* leads to significantly reduced citrus canker symptoms in two citrus species, grapefruit (*Citrus* × *paradisi*) [100] and Wanjincheng orange (*Citrus sinensis* Osbeck) [99].

### CRISPR-mediated resistance against fungi

Fungal pathogens are causal agents of around 30% of emerging plant diseases [108] and infect numerous economically important food crops (Table 1). CRISPR systems have been used to target and disrupt the plant *S* genes for fungal pathogen susceptibility. Barley *Mildew resistance locus O* (*Mlo*) encodes a membrane-associated protein that is essential for fungal pathogen penetration of the host epidermal cells [109], and multiple studies indicated that mutation of *Mlo* triggers plant immunity against powdery mildew [110–112]. Mutating *Mlo* via CRISPR-Cas9 also conferred powdery mildew resistance in wheat [30, 88, 92] and tomato non-transgenic systems [88]. Another powdery mildew *S* gene, *Edr1* (*Enhanced disease resistance 1*), which encodes a Raf-like mitogen-activated protein, has also been targeted via CRISPR-Cas9, resulting in a significant reduction in powdery mildew in wheat [93]. Recently, CRISPR-mediated targeting of the *Powdery mildew resistance 4* (*Pmr4*) *S* gene, whose resistance mechanism is not completely understood, has been demonstrated to cause a significant reduction of powdery mildew disease symptoms in tomato [83].

CRISPR has been used for gene disruption in rice targeting *OsERF922*, which encodes a key ethylene-responsive factor that is involved in the modulation of biotic stress response and is a negative regulator of blast resistance in rice. Single or multiple sgRNAs generated *OsERF922* mutant rice lines that showed tolerance against rice blast, with significantly decreased blast lesions compared to the controls [86]. Prihatna et al. used CRISPR to study the *reduced mycorrhizal colonization* (*rmc*) tomato mutant lines, which contain a chromosomal deletion affecting five genes that makes the plants susceptible to fusarium wilt. CRISPR-Cas9-mediated knock-out and complementation of one of the five genes, *Solyc08g075770*, indicated that it is involved in this fusarium wilt tolerance [85]. Wang et al. used CRISPR to target and disrupt a transcription factor gene, *VvWRKY52*, with a critical role in plant response to biotic stress in the grape (*Vitis vinifera*) cultivar Thompson Seedless and observed increased resistance against the fungal pathogen *Botrytis cinerea* [87]. Likewise, Zhang et al. demonstrated that CRISPR-mediated disruption of the gene encoding tomato mitogen-activated protein kinase 3 (SlMAPK3), which regulates the accumulation of reactive oxygen species (ROS), generated tomato plants resistant to *B*. *cinerea* [84].

### CRISPR-mediated resistance against oomycete

Apart from the major viral, bacterial, and fungal pathogens, CRISPR has also been used to address other biotic stresses, such as oomycete infection (Fig. 1). *Phytophthora palmivora* is a destructive oomycete pathogen of papaya; the CRISPR-Cas9 system was used to develop papaya plant mutant for a functional cysteine protease inhibitor (PpalEPIC8), which led to increased resistance against *P*. *palmivora* [101]. Likewise, resistance has been engineered against another oomycete pathogen, *Phytophthora tropicalis*, in *Theobroma cacao*, the tropical tree that produces cocoa beans [102].

## Limitations and future prospects

CRISPR is being increasingly used to introduce desired traits, including disease resistance, in numerous economically important crop species. Several independent studies have demonstrated successful CRISPR-mediated engineered resistance and, in some cases, broad-spectrum resistance against multiple pathogens (Table 1) [50, 51]. Moreover, these demonstrations of CRISPR-mediated disease resistance have not been limited only to the laboratory or greenhouse: several CRISPR crop varieties are in the pipeline for commercialization and at least one product, false flax (*C*. *sativa*) with enhanced omega-3 oil, is reaching the market in record time in the USA [25]. This is an indication that CRISPR crops and their products will reach consumers in the near future, demonstrating that the exciting applications we have discussed here have great potential in the development of future commercial crop varieties.

There have been several recent developments in the CRISPR technology that can be directly implemented in disease-resistant crop production: for example, generating gene-edited dicotyledonous plants through de novo meristem induction and eliminating time-consuming tissue culture steps [113], using temperature-tolerant CRISPR/LbCas12a to increase the targeting and efficiency [114], enabling large DNA insertions (up to 2 kb) with precision in rice [115], and applying heat-inducible CRISPR system to increase the efficiency of gene targeting in maize [116]. Chromosome engineering in crops is another exciting recent development enabling controlled restructuring of plant genomes [117] and breaking genetic linkage via somatic chromosome engineering [118]. Taken together, these developments would further streamline the transfer of resistance genes to elite cultivars.

Notably, the most economically important plant virus diseases are caused by geminiviruses, and all studies to date of CRISPR-mediated geminivirus resistance have used direct virus DNA targeting. This approach has its limitations, however, owing to the possibility of virus escape and generation of resistance-blocking strains (see the “CRISPR-mediated resistance against plant viruses: targeting virus genomes” section). The most probable solution is the utilization of host susceptibility factors involved specifically in the plant-geminivirus interaction. However, whereas there are well-characterized *S* genes for other pathogens, such as *Mlo* for powdery mildew and *eIF4E* for potyviruses, such high-performance, precise S genes are not currently available for geminiviruses. Considerable work has been done to understand the process of geminivirus infection, and several relevant plant genes have been identified; these comprehensive review articles summarize host susceptibility factors that may be potential target *S* genes for engineering geminivirus resistance, an important strategy to be pursued in the future [119–122].

Given that the CRISPR technology was developed very recently, and it takes several years to develop commercial crop varieties and move them through the standard regulatory procedures, there is a need to revise the regulatory timeline. Moreover, the current legal framework within the EU regulates CRISPR crops as GM crops, even when the products are transgene-free and contain no foreign DNA [123, 124]. These regulatory frameworks increase the time and cost of variety development. Scientists and policymakers need to work together and devise comprehensive plans for CRISPR crops integration. Excellent examples of such efforts include a science-based regulatory framework designed for engineered crops [125] and a GMO opt-in mechanism laid out for the EU [126].

Apart from improvements to the regulatory and policy environment, several technological improvements are also needed to facilitate the development and testing of CRISPR crops. A technological bottleneck to CRISPR in plants is the low innate HDR efficiency, which hinders several intended applications, such as gene replacement and large chromosomal deletions. Although new strategies are being developed to improve HDR efficiency in plants [57, 58, 127], this hurdle currently makes gene-insertion applications in plants challenging. Developing novel methods for the delivery of the genome engineering machinery into germline cells will unlock the potential of this technology to generate foreign DNA-free edited genomes. It will enable engineering wild relatives or germplasm currently used in agriculture but recalcitrant for transformation. Finally, extensive field trials are needed to test the performance of these crops, at least for sustained resistance, and also for other productivity traits that may be compromised if, for example, a multifunctional *S* gene is disrupted.

## Supplementary Information

**Additional file 1.** Review history.
