# Supplementary material for: Engineering crops of the future: CRISPR approaches to develop climate-resilient and disease-resistant plants
Source: Genome Biol. 2020 Nov 30;21:289. doi: 10.1186/s13059-020-02204-y (PMC7702697; doi:10.1186/s13059-020-02204-y)
Supplement: Supplementary file 1 — Additional file 1. Review history. [file 13059_2020_2204_MOESM1_ESM.docx]

Review History

**First round of review**

**Reviewer 1**

**Comments to author:**

I read manuscript of Zaidi et al. with great interest. The review sums up the most recent development of an exploding field in a proper way. It really gives a good overview.  It digs deep into the topic but the reader is not lost in details. As this review  has a clear focus on the production of disease resistant crop plants by the use of CRISPR/Cas it is in a positive way different to the many other reviews that are popping out everywhere since years on the use of CRISPR/Cas in plants . It will be of great interest to a wide audience. I whole-heartily recommend publication
There are few smaller points where would suggests some improvements for the authors:
Page 3; 43:
replicons instead of "replicaons"
Page 12. 48 ff Prospects:
Chromosome engineering in crops (Nat Commun. 2020 doi: 10.1038/s41467-020-18277-z; Nat Plants. 2020 10.1038/s41477-020-0663-x) should also be considered in future as an option for the transfer of resistance genes to elite cultivars
I also would suggest that the authors cite some more very recent papers on the improvement of GT in plants (Nat Biotechnol. 2020 10.1038/s41587-020-0581-5; https://doi.org/10.1016/j.molp.2020.06.008; https://doi.org/10.1111/pbi.13426)

Figure 1: T
To my knowledge no study was published till now that a viroid infection can be blocked with Cas13 (nothing is mentioned in the text).

**Reviewer 2**

**Comments to author:**

In this review, the authors presented a landscape of the studies using CRISPR associated approaches to generate disease resistant crops. The authors comprehensively and systematically summarized the strategies either generating nonsense mutations or regulatory expression on susceptible genes, sense mutations on resistant gene, or targeting the pathogen like virus genomes. It also comprehensively included the recent researches on pathogens such as viruses, fungi and bacteria among diverse host plant species. The scope of the literature basically covered the existing most of the important research and reports. Thus, in my opinion, this paper is informative and of reference value. I here just have some little suggestions as below.
i)     As on a pathogen genome targeting strategy using CRISPR/Cas systems, they were required the exogenous elements of genome editing machinery presented forever in recipient plant genome. Thus, it loses the advantage of DNA free in end-products such as other S or R gene editing. The author can appropriately compare the advantages and disadvantages of the strategy with the formerly existed RNAi strategy and provide some specific discussions and suggestions in more detail for readers.
ii)     In title, "CRISPR-mediated" could be modified. There is no "CRISPR" anymore. We just used the underlined CRISPR mechanism.
iii)      The language of this article could also be polished by someone who is one of a native English speakers. It will be helpful for article reading for readers.
In summary, I suggest that this paper could be acceptable for publication on Genome Biology after some minor revisions.

**Authors Response**

**Point-by-point responses to the reviewers’ comments:**

development of an exploding field in a proper way. It really gives a good overview. It digs deep into the topic but the reader is not lost in details. As this review has a clear focus on the production of disease resistant crop plants by the use of CRISPR/Cas it is in a positive way different to the many other reviews that are popping out everywhere since years on the use of CRISPR/Cas in plants. It will be of great interest to a wide audience. I whole-heartily recommend publication

Re: We thank the reviewer for critically reviewing our article and providing encouraging feedback. The minor points raised by the reviewer have been addressed below.

There are few smaller points where would suggests some improvements for the authors:

Page 3; 43: replicons instead of "replicaons"

Re: Corrected.

Page 12. 48 ff Prospects:

Chromosome engineering in crops (Nat Commun. 2020 doi: 10.1038/s41467-020-18277-z; Nat Plants. 2020 10.1038/s41477-020-0663-x) should also be considered in future as an option for the transfer of resistance genes to elite cultivars

I also would suggest that the authors cite some more very recent papers on the improvement of GT in plants (Nat Biotechnol. 2020 10.1038/s41587-020-0581-5; https://doi.org/10.1016/j.molp.2020.06.008; https://doi.org/10.1111/pbi.13426)

Re: Good suggestions, thank you. A brief discussion of these papers, and the corresponding references, have been added in the revised manuscript. The revised section reads as follows:

There have been several recent developments in the CRISPR technology that can be directly implemented in disease-resistant crop production. For example, generating gene-edited dicotyledonous plants through de novo meristem induction and eliminating time-consuming tissue culture steps [108], using temperature‐tolerant CRISPR/LbCas12a to increase the targeting and efficiency [109], enabling large DNA insertions (up to 2 kb) with precision in rice [110], and applying heat-inducible CRISPR system to increase the efficiency of gene targeting in Maize [111]. Chromosome engineering in crops is another exciting recent development enabling controlled restructuring of plant genomes [112] and breaking genetic linkage via somatic chromosome engineering [113]. Taken together, these developments would further streamline the transfer of resistance genes to elite cultivars.

References

108.	Maher, M.F., et al., Plant gene editing through de novo induction of meristems. Nat Biotechnol, 2020. 38(1): p. 84-89.

109.	Merker, L., et al., Enhancing in planta gene targeting efficiencies in Arabidopsis using temperature-tolerant CRISPR/LbCas12a. Plant Biotechnol J, 2020.

110.	Lu, Y., et al., Targeted, efficient sequence insertion and replacement in rice. Nat Biotechnol, 2020.

111.	Barone, P., et al., Efficient Gene Targeting in Maize Using Inducible CRISPR-Cas9 and Marker-free Donor Template. Mol Plant, 2020. 13(8): p. 1219-1227.

112.	Beying, N., et al., CRISPR-Cas9-mediated induction of heritable chromosomal translocations in Arabidopsis. Nat Plants, 2020. 6(6): p. 638-645.

113.	Schmidt, C., et al., Changing local recombination patterns in Arabidopsis by CRISPR/Cas mediated chromosome engineering. Nat Commun, 2020. 11(1): p. 4418.

Figure 1:

To my knowledge no study was published till now that a viroid infection can be blocked with Cas13 (nothing is mentioned in the text).

Re: Yes, the reviewer is correct––that is why we placed a question mark in the figure, but we thank the reviewer for pointing out that this requires additional clarification. This has been clarified in the revised manuscript.

**Reviewer #2:** In this review, the authors presented a landscape of the studies using CRISPR associated approaches to generate disease resistant crops. The authors comprehensively and systematically summarized the strategies either generating nonsense mutations or regulatory expression on susceptible genes, sense mutations on resistant gene, or targeting the pathogen like virus genomes. It also comprehensively included the recent researches on pathogens such as viruses, fungi and bacteria among diverse host plant species. The scope of the literature basically covered the existing most of the important research and reports. Thus, in my opinion, this paper is informative and of reference value. I here just have some little suggestions as below.

Re: We thank the reviewer for critically reviewing our article and providing encouraging feedback. The minor points raised by the reviewer have been addressed below.

i)	As on a pathogen genome targeting strategy using CRISPR/Cas systems, they were required the exogenous elements of genome editing machinery presented forever in recipient plant genome. Thus, it loses the advantage of DNA free in end-products such as other S or R gene editing. The author can appropriately compare the advantages and disadvantages of the strategy with the formerly existed RNAi strategy and provide some specific discussions and suggestions in more detail for readers.

Re: Thank you for this very good suggestion. Indeed, it’s very tempting to discuss the parallels between RNAi and CRISPR, and address the lessons learned from the RNAi field trials. However, this goes beyond the scope of this article. We even had to leave out some of the outstanding CRISPR crop papers to stick to the “disease-resistance” aspect only.

ii)	In title, "CRISPR-mediated" could be modified. There is no "CRISPR" anymore. We just used the underlined CRISPR mechanism.

Re: The title has been modified for clarity.

iii)	The language of this article could also be polished by someone who is one of a native English speakers. It will be helpful for article reading for readers.

Re: Thank you for the suggestion. We (and a professional science editor) have revised the manuscript critically and tried to improve the language where needed for the purpose of clarity.

In summary, I suggest that this paper could be acceptable for publication on Genome Biology after some minor revisions.
